# Variability in Sleep Timing and Dietary Intake: A Scoping Review of the Literature

**DOI:** 10.3390/nu14245248

**Published:** 2022-12-09

**Authors:** Adriana Rusu, Dana Mihaela Ciobanu, Georgeta Inceu, Anca-Elena Craciun, Adriana Fodor, Gabriela Roman, Cornelia Gabriela Bala

**Affiliations:** 1 Department of Diabetes and Nutrition Diseases, Iuliu Hatieganu University of Medicine and Pharmacy, 400006 Cluj-Napoca, Romania; 2 Diabetes Center, Emergency Clinical County Hospital Cluj, 400006 Cluj-Napoca, Romania

**Keywords:** sleep variability, social jetlag, dietary intake

## Abstract

The objective of this scoping review was to summarize previous studies which examined the effect of day-to-day variability in sleep timing and social jetlag (SJL) on dietary intake. A systematic literature search was conducted in PubMed, Embase, and Clarivate Analytics Web of Science and we identified 22 records. No difference in caloric and macronutrient intake between SJL groups was observed in studies that enrolled healthy young adults. However, studies that enrolled participants with obesity and obesity-related chronic conditions reported a higher caloric intake and a higher intake of carbohydrates, total fat, saturated fats, and cholesterol in participants with SJL than in those without. Most studies reported a lower quality of diet, a delayed mealtime, and eating jetlag in participants with SJL vs. those without SJL. No correlation of day-to-day variability in sleep timing with average caloric intake was observed, but bed-time variability was negatively associated with diet quality. Methodological issues have been identified in sources assessed including study design, power calculation, population enrolled, and tools/metrics used for sleep timing variability assessment. Future well powered longitudinal studies, with clear protocols, standardized metrics, including all age groups from general population are needed to clarify the dietary intake consequences of variability in sleep timing.

## 1. Introduction

Sleep health is an essential element of cardiovascular (CV) and general health. The construct of Life’s Simple 7, which included seven health behaviors and health factors—diet, physical activity, nicotine exposure, body mass index, blood lipids, blood glucose, and blood pressure—associated with CV disease (CVD)-free survival, total longevity and quality of life, was recently updated by the American Heart Association to Life’s Essential 8, including sleep as the new health behavior in the construct [1]. Sleep is one of the foundational elements in human biology and affects numerous physiological functions. Sleep health is defined by several characteristics including sleep duration, timing, regularity, efficiency, satisfaction, and impact on daytime alertness [2].

Much of the existing research is focused on the relationship between sleep duration and CVD risk and all-cause and CV mortality [3], as well as the impact of sleep duration on various health risk factors including obesity [4]. Variability in sleep timing has been recently emphasized as a link between sleep health indicators and risk of illnesses. Social jetlag (SJL), defined as variation of wake up and sleep onset time between weekdays and weekends [5], is a frequent form of circadian rhythm disruption with 70% of students and workers experiencing ≥1 h of SJL and almost half of them experiencing ≥2 h [6]. SJL has been associated with several related risks for human health such as impaired sleep and cognitive performance, obesity, diabetes, negative CV outcomes, and psychiatric disorders [7]. Even more, the day-to-day sleep variability may also play a role in health and disease [8] which is less studied than the role of SJL [9].

The relationship between variability in sleep timing and negative health outcomes could be explained through several mechanisms such as alterations in the hypothalamic–pituitary–adrenal axis and autonomic nervous system functioning, alterations in circadian processes leading to subclinical inflammation, dysregulated patterns of cortisol release, insulin resistance, and suppression of nocturnal melatonin secretion, which in turn will decrease its antioxidant, anti-inflammatory effects, and its inhibitory effect on leukocytes’ adherence to endothelial cells [9]. Other mechanisms could be involved, as behavioral processes are also altered (e.g., taking naps, drinking caffeine or alcohol, using sleeping medications, and having a poorer diet). Dietary changes are of particular interest in explaining higher risk for metabolic adverse outcomes including weight gain and available conflicting research reports regarding overall caloric intake, dietary quality, or pattern of meal timing.

No systematic approach is currently available for measuring sleep timing variability/sleep timing regularity and several metrics have been proposed. Traditional metrics that include intra-individual standard deviation (StDev), interdaily stability (IS), and SJL compare daily sleep–wake patterns to an individual’s average pattern [10]. SJL is considered a measure of weekly sleep variability [5], while StDev and IS measure sleep variability across multiple days [10]. More recently, two metrics that assess variability in sleep–wake patterns between consecutive days have been proposed—composite phase deviation (CPD) and sleep regularity index (SRI) [10]. Other metrics used include the coefficient of variation, intradaily variability or statistical modeling [10]. Several variables have been shown to influence the performance of these metrics and the results of studies reporting sleep-timing variability, including naps, sleep fragmentation, length of data collection, and sample size [10]. Thus, these aspects must be accounted for when deciding what type of metric is used to assess sleep variability in research studies.

No scoping or systematic review is currently available on the effect of sleep pattern variability on dietary intake. There are reviews focusing on the relationship between sleep duration, quality, and diet or between SJL and health outcomes [11,12,13].

Therefore, the objective of this scoping review was to systematically evaluate results from previous research which examined the effect of SJL and day-to-day variability in sleep timing on dietary intake, assessed as caloric intake, food preferences, dietary patterns, or mealtimes, to critically discuss the methodology used and its potential impact on the results, as well as to identify existing gaps in the knowledge.

## 2. Materials and Methods

A scoping review protocol compliant with Preferred Reporting Items for Systematic Reviews and Meta-Analyses (PRISMA) extension for Scoping Reviews [14] and Joanna Brigg’s Institute Reviewer’s Manual for Scoping Reviews [15] was developed.

### 2.1. Eligibility Criteria

We included cross-sectional studies, prospective/retrospective observational studies, and interventional trials performed in adults (>18 years of age), which reported variability in sleep timing (day-to-day sleep variability or weekday to weekend variability in sleep timing, also known as SJL) and dietary intake and were published either as full-length articles or conference abstracts. To be included in this scoping review, the variability in sleep timing had to be assessed by questionnaires, sleep diaries or actigraphy, and dietary intake by direct observation, food frequency questionnaires or food diaries, visual analog scales (VAS) for food preferences and other dietary intake-specific questionnaires.

We excluded publications in other languages than English, case reports, commentaries, personal opinions, review articles, meta-analyses and book chapters, studies that enrolled children or adolescents, persons working on night shifts, and studies performed in other species than humans.

### 2.2. Information Sources and Literature Search

To identify studies of interest, the literature search was conducted in 3 databases (PubMed, Embase, and Clarivate Analytics Web of Science) for articles published from the inception dates until the 31st of December 2021.

The search queries used are provided in Appendix A. Abstracts and proceedings, considered as gray literature, were identified during Embase search. References of reviews [11,12,13] were screened for relevant studies not identified during database searches.

### 2.3. Selection of Sources of Evidence

Investigators working in pairs screened the identified records. The search results were exported from databases, duplicate reports were removed, and the titles and abstracts of the remaining reports were screened to exclude studies that did not meet the eligibility criteria. Copies of all remaining studies were retrieved and reviewed in full to identify studies assessing dietary intake according to sleep variability and fulfilling the inclusion and exclusion criteria presented below. During the study selection step, any disagreement between the investigators was resolved by discussions and consulting a review author not actively involved in the study selection.

### 2.4. Data Charting Process, and Data Items

After an agreement was reached on all articles, data were extracted using an Excel-based data extraction form. The following information was extracted whenever available: author, year of publication, study design, type of variability in sleep timing reported (day-to-day, SJL), method of assessment of sleep variability, sample size, age, gender, weight status, other metabolic diseases, caloric intake, intake of macronutrients (carbohydrates, proteins, lipids, cholesterol, saturated fats), intake of food groups or dietary patterns, food preferences/appetite for certain foods, and meal timing.

### 2.5. Critical Appraisal of Individual Sources of Evidence

All studies included in this review were observational ones. Thus, their quality was assessed with the National Heart, Lung, and Blood Institute’s Quality Assessment Tool [16]. Due to the limited number of available sources and the lack of a previous systematic or scoping review on this subject we chose to also explore their quality.

### 2.6. Synthesis of the Results

A narrative synthesis of the results was performed. Characteristics of studies included were categorized and summarized according to the type of variability in sleep timing (day-to-day or SJL), including populations, study designs, measures used for assessing sleep patterns variability and dietary intake and the main findings of interest.

## 3. Results

### 3.1. Selection of Sources of Evidence

**Figure 1:**
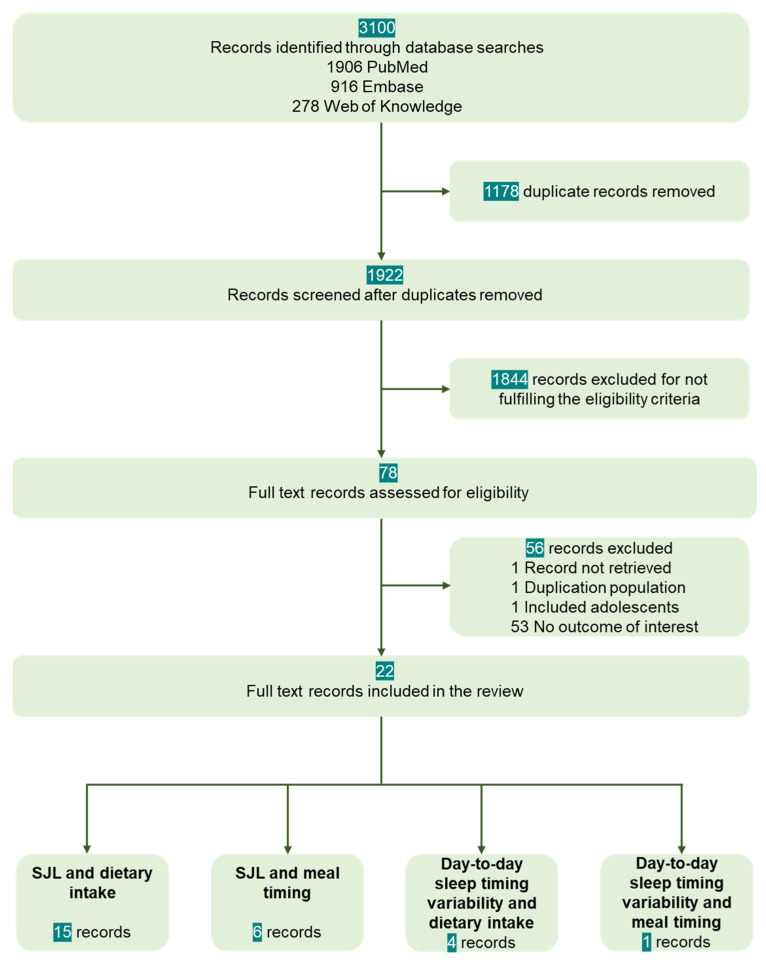
Records selection process.

Our search strategy identified 3100 database records. After removing 1178 duplicates, the titles and abstracts of 1922 records were screened for eligibility criteria and outcomes of interest. Of these, 1844 were removed due to the lack of assessment of any circadian rhythm or sleep parameters, lack of assessment of any dietary intake or mealtimes, the inclusion of children or adolescents, shift workers, animal studies, records in other languages than English, or record type review, case presentation, comment, or book chapter. The full text of 78 records was retrieved for further assessment. Of these, 1 could not be retrieved, 1 presented results of the same population as an article already included, and 53 did not fulfill eligibility criteria (the majority did not assess any circadian rhythm or sleep parameters, or any dietary intake or mealtimes). Thus, 22 records (20 full-text articles and 2 conference abstracts) were included in the review (Figure 1).

### 3.2. Study Characteristics

All studies included were observational, 21 with a cross-sectional design [17,18,19,20,21,22,23,24,25,26,27,28,29,30,31,32,33,34,35,36,37] and 1 with a longitudinal design [38]. Seventeen studies had listed, among the objectives, the assessment of the influence of sleep–wake rhythm (SJL or day-to-day sleep timing variability) on dietary intake, appetite, preference, or meal timing (our outcomes) [18,19,20,23,24,25,27,28,29,30,31,32,34,35,36,37,38]. One study assessed the association of SJL with consumption of stimulants, including sugar-sweetened beverages, without assessing other components of the diet [22]. Four studies reported the association of SJL with dietary intake or SJL with meal timing as incidental findings [17,21,26,33].

Sample sizes varied from small (<100 participants in seven studies) [23,24,25,28,31,34] to large samples of thousands of participants [19,21,30,33,35,36]. Sample size or power calculation was not performed in 72.7% of the included studies [18,19,20,21,22,23,24,25,27,28,29,30,32,33,34,37]. In four additional studies, sample size was assessed but not for the association of SJL with dietary intake [17,26,35,36].

All studies included adults, and 11 of them included only young adults (eight included only highly educated young adults) [17,18,20,22,24,28,29,31,32,33,34]. A general population sample or healthy adults were included in 16 studies [17,18,19,20,22,23,24,28,29,30,31,32,33,34,35,36], while 5 studies included adults with obesity and obesity-related chronic diseases (including prediabetes and type 2 diabetes) [21,25,26,27,38].

### 3.3. Assessment of Sleep Timing Variability and Dietary Intake

In most of the studies, the variability of sleep timing was assessed by questionnaires. Seven day sleep logs were used in one study [25] and wrist actigraphy in four studies (one assessing SJL [31] and three assessing day-to-day variability of sleep timing [18,34,37]). As metrics, weekly sleep pattern variability was assessed using SJL in 17 studies [17,20,21,22,23,24,25,26,27,28,29,30,31,32,33,35,38]. In two studies SJL was calculated using time in bed [30,32] and in one study using sleep duration [35], for two studies the methodology for calculation was not disclosed [23,25] and for twelve studies the mid-sleep time was used [17,20,21,22,24,26,27,28,29,31,33,38].

Daily sleep timing variability was assessed using StDev in two studies [18,37], with he weighted average of weekly bedtimes variability dichotomized between lower and higher variability according to scores obtained with the Sleep Timing Questionnaire in one study [19] and with a self-assessment of sleep onset/offset variability in one study [36]. In one study the metrics used and the methods for sleep onset/offset calculations were not disclosed [34]. 

Dietary intake was assessed by 24 h food recall in seven studies [20,21,23,26,27,28,38], by FFQ in five studies [17,29,30,31,36], by food diary in one study [35], and by an ad libitum test meal in one study [24]. Additionally, diet quality assessment tools were used in two studies [19,32], time-stamped photographic food records for 3 days in one study [25], and 7 day food diaries in two studies [18,34]. Appetite was assessed using VAS [24,28] while the timing of meal intake was assessed by 24 h food recall, meal timing journals and questionnaires on eating time [33,37].

### 3.4. SJL and Dietary Intake

Fifteen studies examined the association between SJL and dietary intake. Caloric and/or macronutrient intake was reported in 9 studies [20,24,25,26,27,28,30,31,38], diet quality and food items in 11 studies [17,20,22,23,25,27,29,30,31,32,35] and prospective appetite in 2 studies [24,28] (Table 1).

### 3.5. SJL and Meal Timing

No difference in caloric and macronutrient intake between groups with or without SJL was observed in cross-sectional studies that enrolled a general population sample, healthy young adults, or patients with type 2 diabetes and in which the estimation of caloric intake was performed using only one 24 h food recall [24,25,28]. However, studies that enrolled participants with obesity and obesity-related chronic conditions reported a higher caloric intake and a higher intake of carbohydrates and total fat, mono- and polyunsaturated fats, saturated fats, and cholesterol in participants with SJL as compared to those without [25,27,38]. One study examined changes in caloric intake between workdays and free days and reported a significantly higher caloric intake and a higher intake of total fat, saturated fat, and MUFA as well as a lower fiber intake during free days than during workdays in participants with SJL but not in those without SJL [20].

Most studies reported a lower quality of diet in participants with SJL vs. those without SJL [20,35] and lower adherence to the Mediterranean diet [32] or a healthy Nordic diet [30]. In the study of Almoosawi et al. [35] an inverse u-shaped association of SJL with diet quality was observed with the inflexion point at 1 h and 45 min; below this point the diet quality increased in parallel with SJL duration, while above this point the diet quality decreased. SJL was also associated with a lower consumption of total fruits, berries, vegetables, whole grains, beans, and milk [29,30,31,32] but more alcohol, sugar, or sugar-sweetened beverages, meat, and eggs [20,27,30,31]. Of note, two studies reported no difference in diet quality between SJL and no SJL [17]; the largest one enrolled only young, highly educated women [17], and the other study enrolled a very small sample and the full list of inclusion/exclusion criteria could not be assessed [25].

Prospective appetite was assessed using VAS in two small sample-size studies [24,28]. A higher perceived appetite was reported in fasting conditions for vegetables, pork, poultry, fish, eggs, milk, and dairy products in participants with SJL as compared to those without SJL [28]. Furthermore, higher ratings of hunger and prospective food intake after the meal was reported in participants with SJL > 1 h than in the SJL ≤ 1 h group despite similar caloric intake [24]. Post-meal satiety quotient (mean value of the satiety quotients for hunger, prospective food intake, satiety, and fullness) was significantly lower in participants in the 1 < SJL ≤ 2 h and SJL > 2 h groups (1.3 and 1.7 times, respectively) compared to those in the SJL ≤ 1 h group (*p* < 0.010) [24].

The relationship between SJL and meal timing was reported in six cross-sectional studies [20,21,27,28,29,33] (Table 2). A delayed mealtime (breakfast, lunch and/or dinner) and eating jetlag was reported in those with SJL, in all studies that assessed these parameters [21,27,28,29,33]. Also, a higher frequency of snacking after dinner and a later time of this snack as well as a higher caloric intake after 9 p.m. and a higher proportion of calories consumed after 9 p.m. were observed in persons with SJL than in those without [27,28].

In a study that assessed the change in meal timing in free days compared to workdays, Bodur et al. [20] reported the delayed timing of all meals irrespective of SJL presence with a significantly longer delay in breakfast timing in those with SJL than in those without.

With regards to eating window, it was assessed in two studies. In the first one, Mota et al. [27] reported overall a longer eating window in patients with obesity-related chronic diseases and SJL. In the second study, Bodur et al. [20] found a shorter eating window during free days in those with SJL as compared to those without SJL (8:42 vs. 9:00).

### 3.6. Day-to-Day Variability in Sleep Timing, Dietary Intake, and Meal Timing

Four cross-sectional studies assessed the day-to-day variability in sleep timing and dietary intake [18,19,34,36] (Table 3). In a study performed in young and middle-aged adults from US general population, Hooker et al. [18] found no significant correlation between the variability in sleep timing, as assessed by StDev, with average caloric intake or with variability in caloric intake. In another study using 7 days of wrist actigraphy for the assessment of the variability in sleep timing and food diaries for 7 days, Chan et al. [34] reported that young adults with bedtime variability over 90 min consumed high palatability foods at breakfast less frequently than those with bedtime variability < 90 min; no difference was observed for lunch and dinner. However, in this later study the metric used for sleep time variability was not disclosed.

The largest studies were performed in Australian and Japanese populations. In the study performed in Australian middle-aged adults and elderly, Duncan et al. [19] reported that bed-time variability was negatively associated with diet quality, independent of waking time variability, usual bedtime and waking time, age, gender, smoking status, number of health conditions, work schedule, body mass index (BMI) and days of insufficient sleep. In this study the sleep variability was assessed using a weighted average of variability of sleep timing (sleep timing questionnaire) [19]. In the other study performed in Japanese adults, sleep pattern variability was self-declared for the previous year and Yamaguchi et al. [36] showed that poor sleep regularity was associated with low protein intake, high carbohydrate intake and a variability of staple foods consumption between breakfast, lunch, and dinner.

The association of day-to-day variability in sleep timing with meal timing was assessed and confirmed in only one study that enrolled patients with eating disorders and for which sleep variability was measured as StDev of center of daily inactivity [37].

### 3.7. Critical Appraisal of the Individual Sources of Evidence

**Figure 2:**
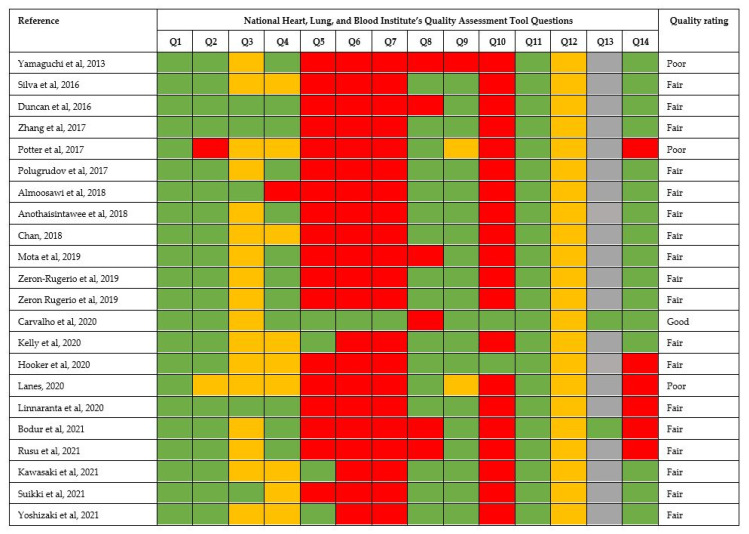
Quality assessment [17,18,19,20,21,22,23,24,25,26,27,28,29,30,31,32,33,34,35,36,37,38].

By the National Heart, Lung and Blood Institute’s Quality Assessment Tool, one study was rated as good [38], three studies as poor [23,25,36] and eighteen as of fair quality [17,18,19,20,21,22,24,26,27,28,29,30,31,32,33,34,35,37] (Figure 2).

## 4. Discussion

### 4.1. Main Findings Related to Research Question and Discussion of Specific Findings

This scoping review is the first one in the literature to comprehensively assess the available evidence on the effect of weekly and day-to-day variability in sleep timing on dietary intake, appetite, and mealtimes. Limited evidence from the studies discussed in this scoping review suggests that variability in sleep timing (both SJL and day-to-day variability) are associated with a less healthy diet [19,20,30,32,35], characterized by a lower consumption of fruits, vegetables, whole grains, beans, and milk [29,30,31,32] and a higher intake of sugar, or sugar-sweetened beverages, and meat compared with persons without SJL [19,27,30,31]. Also, very limited evidence available suggests that persons with SJL have a higher perceived appetite for energy-dense foods, higher ratings of hunger, and prospective food intake after the meal and lower post-meal satiety quotient [24,28].

These results are in line with previous exploratory studies that showed that acute circadian dysregulation induced by alterations in the sleep–wake schedule is associated with increased ratings of hunger, prospective food consumption, and a desire to eat savory foods [39,40]. Interestingly, chronic circadian dysregulation in exploratory conditions was associated with decreased energy expenditure and decreased hunger and appetite for various foods [41]. The mechanism regulating unhealthy food preferences in SJL and day-to-day variability in sleep timing is unknown. Preliminary observational data from functional magnetic resonance scans suggest an increased activation of brain regions associated with reward in persons with SJL independent of sleep duration [42]. Food intake is regulated by (1) the homeostatic system, controlled by structures located in the hypothalamus and the brainstem which regulates the need for food intake [43,44,45], and (2) the reward system, consisting of structures from the mesolimbic pathway and having dopamine as the main neurotransmitter [46,47], which regulates the hedonic aspects of feeding and the pleasure to eat. In addition to the homeostatic and the hedonic control of eating, the circadian system regulates the time of eating [48] and circadian dysregulation may result in changes of eating behavior [49]. It has been shown that caloric intake and food preferences have circadian rhythms [50,51,52] and are controlled by the circadian system through projections from the suprachiasmatic nucleus master clock to hypothalamic nuclei regulating homeostatic feeding behavior [53,54,55] and to the striatum regulating hedonic food intake [49]. Thus, the circadian system plays a key role along with the homeostatic and the hedonic systems in the regulation of food intake. Other mechanisms potentially associated with the unhealthy food choices observed in those with SJL and day-to-day variability in sleep timing are the use of certain foods, such as sugar-sweetened beverages or sugar-containing foods, being stimulants allowing subjects prone to sleep loss to stay alert [22,56]. Additionally, the preference for certain foods may be influenced by social factors and limited time available for sourcing and eating certain foods due to work schedules and daily habits, as reported by shift workers [56].

In addition to an unhealthy diet, studies that enrolled participants with obesity and obesity-related chronic conditions included in this review reported a higher caloric intake and a higher intake of carbohydrates and total fat, mono- and polyunsaturated fats, saturated fats, and cholesterol in participants with SJL as compared to those without [25,27,38]. Despite the poorer quality of diet and an increased self-rated appetite for unhealthy foods, no difference in caloric and macronutrient intake between groups with or without SJL was observed in studies that enrolled the general population and healthy young adults [24,26,28]. This could be explained by the use of 24 h dietary recall only for the previous day which may not adequately reflect the usual caloric and macronutrient intake. In addition, many studies that showed no difference in caloric intake included participants with higher education and good health literacy on the link between diet and health which might mitigate SJL-related food behavior. This latter hypothesis is supported by our previous observation on higher cognitive restraint scores in highly educated subjects with SJL. In these subjects, caloric or macronutrient intake was similar to those of subjects without SJL despite an increased self-rated appetite for energy-dense foods [28]. Additionally, obesity is commonly associated with an increased caloric intake; thus, it remains to be established whether the higher caloric intake in persons with obesity and SJL is a consequence of SJL or just coexisting with SJL in the context of obesity.

Other observations, although arising from limited evidence, point toward a delayed mealtime and eating jetlag [20,21,27,28,29,33] as well as a higher frequency of snacking after dinner [27,28], a higher caloric intake after 09:00 p.m. and a higher proportion of calories consumed after 09:00 p.m. [27,28] in persons with SJL than in those without. Meal timing variability was also reported in persons with day-to-day variability in sleep timing, although the evidence was in only one study available in the literature, performed in patients with eating disorders [37] and thus limiting the generalizability of the results. Caloric intake during inappropriate circadian phases (e.g., during typical sleep time or biological night) has been associated with metabolic changes thought to be responsible for the deleterious effect on health in both animal models and humans—obesity, cardiovascular diseases, and diabetes [7,57,58,59]. In animal models it has been shown that at the same caloric intake and locomotor activity, the weight gain is higher if the food intake occurs during the biological night as compared to the biological day [58,59]. Furthermore, feeding during the biological night resulted in increased weight gain, while restriction of feeding to the biological day prevented this effect [59]. In humans studied in exploratory conditions under a circadian misalignment protocol mimicking the night shift, McHill et al. [57] showed that the thermic effect of food (TEF) and carbohydrate and protein utilization decreased in response to a late-night dinner. Similarly, TEF was significantly lower after a meal consumed during the night (at 01:00 a.m.) as compared to meals consumed during the morning (09:00 a.m.) and afternoon (05:00 p.m.) [60]. TEF accounts for up to 10% of daily energy expenditure; thus, a reduced TFE may promote weight gain if the caloric intake is maintained at the same level [57]. In real-life settings, Baron et al. [61] showed that caloric intake after 08:00 p.m. predicted the BMI even after controlling for sleep timing and duration. Confirming these findings, Gill et al. [62] showed that restricting the food intake to daytime (06:00 a.m. to 06:00 p.m.) resulted in lower caloric intake and weight loss.

### 4.2. Gaps Identified in the Sources Included, Implications for Future Studies

Although data on SJL and its association with dietary intake are accumulating, evidence on the day-to-day sleep timing variability and its relationship with dietary intake is scarce. Only four studies are available on its association with diet quality, and caloric and macronutrient intake [18,19,34,36], and only one study is available that evaluates its correlation with mealtimes [37].

Furthermore, some methodological issues with existing studies must be noted. All studies included [17,18,19,20,21,22,23,24,25,26,27,28,29,30,31,32,33,34,35,36,37] but one [38] were cross-sectional thus making difficult the inference of a causal relationship between sleep timing variability and dietary intake and their effect on health outcomes. For most of the studies assessed, a sample size calculation was not performed [18,19,20,21,22,23,24,25,27,28,29,30,32,33,34,37] or did not assess sleep variability [17,26,35,36] and, thus they may be underpowered to assess the outcomes. Furthermore, in several studies the association of SJL with dietary intake or meal timing was an incidental finding and its was not included among published study objectives [17,21,26,33].

If a consistency was observed in measuring SJL (Munich Chronotype Questionnaire or several items from this questionnaire), for studies assessing the day-to-day variability in sleep timing, no systematic approach has been used and the metrics varied from StDev to self-declared variability and this may limit the accuracy of the results [18,19,34,36,37]. Although StDev is an accepted metric that estimates sleep variability across multiple days it does not consider naps and fragmented sleep and tends to underestimate sleep variability if calculations are based on data collected on ≤7 days [10]. In one of the two studies that assessed sleep variability using StDev, data were collected for 7 days of sleep actigraphy [18]. Thus, the lack of any correlation between sleep variability and caloric intake in this study may be also due to the metric used.

Although SJL was relatively consistently assessed, calculations were performed using time in bed instead of mid-sleep time in 2two studies [30,32], thus ignoring sleep latency. Also, although in the original formula for SJL calculation developed by Wittmann et al. [5] SJL is calculated as the absolute difference between the mid-point of sleep time on free days and workdays, more recently, Jankowski [63] proposed a formula corrected for sleep debt. Debate exists regarding the use of these formulas and there is a need to clarify which metric reflects better the circadian misalignment and is associated with adverse health outcomes [64].

Chronotype influence on the relationship between sleep timing variability and dietary intake was not considered in all but one study, which showed the dietary influence of SJL only in participants with a morning chronotype [30]. SJL is more frequent and greater in evening chronotype individuals who prefer a later bedtime and wake up time [64]. Previous research showed changes in clock gene expression patterns in both evening chronotype and SJL [65] and similar disease risk in humans [64]. Both are age-dependent and vary in parallel—chronotype delays up to the end of adolescence and then advances with age, while SJL is higher during adolescence and then decreases through adulthood until retirement [64]. From these observations arises the question on whether the evening chronotype can be considered a form of SJL. And the answer is most probably ‘no’ as although chronotype is a state of phase of entrainment reflecting the continuous adaptations of circadian system to zeitgeber changes [66,67], it also has a genetic basis [68]. SJL is a metric describing the chronic misalignment between the circadian system and social schedules, results from sleep timing variability between weekdays and weekends and includes a component of sleep debt that may influence associated health risks [64]. Furthermore, SJL is not solely seen in evening chronotypes; due to social pressure, it is observed in morning chronotypes [64]. Thus, studies should explore the chronotype-related differences in sleep and meal regularity.

Given the identified gaps detailed above, well-powered studies are needed to assess whether sleep variability is associated with dietary intake. Even more so, longitudinal studies may help clarify a potential causal relationship between sleep variability and dietary intake and other health issues reported in the literature. Additionally, methodological limitations arising due to the use of unvalidated metrics for the evaluation of day-to-day sleep variability could be overcome by clear protocols, clear definitions of outcomes and metrics used, and choosing validated metrics adapted for the sample size, study length, and pertinent for research question. The inclusion of all age groups and general population, irrespective of comorbidities, without limitation of participants to young, healthy, and highly educated ones is also recommended to avoid negative results biased by health literacy that may influence food choices and food intake. Future research should also clarify whether nutritional changes associated with variability in sleep timing, are associated with poor health outcomes.

### 4.3. Limitations of the Scoping Review

Although this is the first scoping review assessing the published literature on the association between variability in sleep timing and dietary intake, this review has limitations. The narrative synthesis did not allow the weighting of the studies analyzed. The results may be biased by the methodological quality of the studies; most of the studies were observational and the risk of publication bias is high. The results of this review may also be biased by not including studies on this topic indexed in other databases or published in languages other than English. The methodology used varied across the included studies, in terms of design, data collection, or outcomes assessed, thus the development of a meta-analysis was not possible, and a narrative review was carried out.

## 5. Conclusions

The variability in sleep timing, either as SJL or day-to-day variability, promotes an unhealthy diet characterized by lower consumption of fruits, vegetables, whole grains, beans, and a higher intake of sugar and meat. Although limited evidence exists, it seems that persons with SJL may have a higher perceived appetite for energy-dense foods, delayed mealtime, and eating jetlag. Whether these are associated with increased caloric intake, a change in macronutrient intake, and long-term consequences on human health remains unknown. In addition to the limited body of evidence currently available, conclusive statements on the findings of this review are also limited by the methodological variations observed across studies. Future well powered longitudinal studies, with clear protocols, standardized metrics, including all age groups from general population are needed to clarify the dietary intake consequences of variability in sleep timing. Additionally, future research should clarify whether these changes in dietary intake, if they are to be confirmed, are associated with poor health outcomes and whether therapeutic nutritional interventions in persons with circadian and sleep disruption could reverse some of their harmful effects. 

## Figures and Tables

**Table 1:** Studies reporting the association of social jetlag with dietary intake.

Authors, Year	Country	Study Design	Population Sample	Population Type, Gender	Age, Years	SJL Calculation	SJL Assessment Method	Dietary Intake Assessment Method	Main Study Objective	Main Findings	**Limitations**
Bodur et al., 2021 [20]	Turkey	Cross-sectional	710	General population, M/F	19.0–24.0	MSF-MSW	Questionnaire for the previous night	24 h food recall	Energy, macronutrient intake and diet qualities in persons with and without SJL during working vs. non-working days.	Participants with SJL reported during free days vs. workdays: higher energy intake (1531.4 ± 491.3 kcal/day vs. 1443.5 ± 470.6 kcal/day, *p* < 0.001) higher amount of total fat (69.22 ± 27.43 g/day vs. 62.5 ± 26.1 g/day, *p* < 0.001), higher amount of saturated fat (27.6 ± 12.8 g/day vs. 24.0 ± 11.5 g/day, *p* < 0.001) lower intake of fiber (13.7 ± 6.6 g/day vs. 14.9 ± 7.3 g/day, *p* = 0.001) higher MUFA intake (24.1 ± 10.3 g/day vs. 21.9 ± 9.8 g/day, *p* < 0.001) no change in diet quality (HEI 2015 total score 53.06 ± 12.6 vs. 52.6 ± 10.88, *p* = 0.548) Participants without SJL reported during free days vs. workdays no significant change in energy and macronutrients intake (*p* > 0.05) and an increase in diet quality (53.15 ± 10.03 vs. 59.30 ± 10.78, *p* < 0.001) Participants without SJL had significantly higher diet quality (*p* < 0.001) and higher consumption of fruit (*p* = 0.010), whole fruit (*p* = 0.005), whole grain (*p* = 0.008), and milk (*p* = 0.043).	Dietary intake assessed for the 24 h before the study visits. Sleep mid-point assessed only for the night before interview. No power calculation.
Rusu et al., 2021 [28]	Romania	Cross-sectional	80	Healthy normal weight young participants, M/F	31.7 ± 6.7	MSF-MSW	Questionnaire for the previous month	24 h food recall VAS for food preferences	Effect of SJL on perceived appetite, hunger, and ghrelin in healthy subjects in free-living conditions.	No difference was observed between participants with/without SJL for caloric intake (1732.3 ± 642.1 kcal/day vs. 1867.1 ± 752.8 kcal/day) calories from, carbohydrates 51.3 (41.9; 57.7)% vs. 50.7 (42.0; 55.5)% of calories from proteins 15.2 (12.1; 18.7)% vs. 17.0 (13.0; 19.9)% calories from fats 37.2 (30.0; 43.6)% vs. 38.3 (31.6; 41.0)% Participants with SJL reported a higher perceived appetite for vegetables, pork, poultry, fish, eggs, milk, and dairy products than those without SJL.	Dietary intake assessed only for the previous 24 h. No power calculation. Small sample. Statistical significance of differences between groups not tested. Young, healthy, highly educated and normal-weight participants.
Kawasaki et al., 2021 [17]	Japan	Cross-sectional	218	General population, F	19.9 ± 1.6 20.2 ± 1.9	MSF-MSW	MCTQ	54 items FFQ	The association of chronotype with healthful and unhealthful plant-based diet quality	No association of SJL with healthful plant-based diet quality (β = 0.024, *p* = 0.509) nor with unhealthy plant-based diet quality (β = 0.100, *p* = 0.141).	Sample size calculation was performed, but not for SJL. Association of SJL with dietary intake not listed among the study objectives. Only young women enrolled.
Suikki et al., 2021 [30]	Finland	Cross-sectional	6779	General population (FINRISK 2012 and DILGOM 2014), M/F	25–74	Time in bed in weekends—time in bed during weekdays	Questionnaire	131-item FFQ	The association of SJL with diurnal preference, and whether the association of SJL with the quality of the diet and obesity was influenced by diurnal preference.	Participants with a morning chronotype and a SJLsc ≥ 2 h had lower adherence to a healthy Nordic diet than those with a SJLsc < 1 h (BSD scale for adherence 10.5 points vs. 11.8 points, *p* = 0.006), independent of age, sex, educational years, smoking, leisure-time physical activity, and energy intake. Participants with a morning chronotype and a SJLsc ≥ 2 h consumed less fruits, berries, and cereals, but more alcohol, than those with a SJLsc < 1 h (*p* < 0.05) independent of age, sex, educational years, smoking, leisure-time physical activity, and energy intake. Total energy intake did not differ between SJLsc groups according to diurnal preference (*p* = 1.00).	No power calculation.
Yoshizaki et al., 2021 [31]	Japan	Cross-sectional	82	Young adults	19–29	MSF-MSW	7-day wrist actigraphy	135-item FFQ	The associations of chronotype and SJL with the quantity and over- all quality of habitual food consumption	A larger SJL was associated with lower total energy intake (β = −0.094, *p* = 0.013), lower grains consumption (β = −0.143, *p* < 0.001) and greater consumption of sugar and confectioneries (β = 0.231, *p* = 0.010) independent of age, sex, BMI, residential status, and WD and WE total sleep duration.	Young, healthy and highly educated participants.
Carvalho et al., 2020 [38]	Brazil	Prospective, observational	122	Patients with obesity after bariatric surgery, M/F	33.0 (28.0–41.7)	MSF-MSW	Questionnaire SJL evaluated at 3 visits (3 months interval) and a median exposure to SJL calculated	24 h food recall at each visit for workdays and free days	The influence of SJL on anthropometric, metabolic and food intake 6 months after the surgical intervention	Participants with larger SJL exposure vs. small SJL exposure had higher intakes of calories (baseline 2565.6 ± 130.0 kcal/day, 3 months 889.7 ± 34.2 kcal/day, 6 months 1091.9 ± 39.0 kcal/day vs. baseline 2185.0 ± 95.5 kcal/day, 3 months 823.8 ± 32.0 kcal/day, 6 months 937.7 ± 30.6 kcal/day; *p* = 0.001) carbohydrate (baseline 261.4 ± 13.7 g/day, 3 months 70.7 ± 3.7 g/day, 6 months 77.7 ± 3.8 g/day vs. baseline 229.0 ± 9.4 g/day, 3 months 61.0 ± 3.1 g/day, 6 months 68.8 ± 3.5 g/day, *p* = 0.005) and total fat (baseline 116.5 ± 7.4 g/day, 3 months 33.2 ± 1.9 g/day, 6 months 49.5 ± 2.4 g/day vs. baseline 97.3 ± 5.6 g/day, 3 months 32.2 ± 1.7 g/day, 6 months 40.2 ± 1.7 g/day, *p* = 0.007) polyunsaturated fat (baseline 15.9 ± 1.2 g/day, 3 months 5.0 ± 0.3 g/day, 6 months 7.8 ± 0.5 g/day vs. baseline 12.9 ± 0.9 g/day, 3 months 4.5 ± 0.2 g/day, 6 months 5.9 ± 0.3 g/day, *p* < 0.001) and monounsaturated fat (baseline 34.5 ± 1.9 g/day, 3 months 10.2 ± 0.6 g/day, 6 months 15.6 ± 0.9 g/day vs. baseline 30.1 ± 1.9 g/day, 3 months 10.0 ± 0.6 g/day, 6 months 12.8 ± 0.5 g/day, *p* = 0.035)	Dietary intake assessed only for the previous 24 h. Sample selected from a private clinic and included only those with gastric sleeve or Roux-en-Y gastric bypass.
Kelly et al., 2020 [26]	Ireland	Cross-sectional	252	Type 2 diabetes, M/F	61.9 ± 10.5	MSF-MSW	MCTQ	24h food recall	The association of chronotype and/or SJL with glycemic control.	No difference was observed between SJL groups in: daily caloric intake (SJL < 30 min: 1360.19 ± 284.55 kcal, SJL 30–90 min: 1410.52 ± 470.32 kcal, SJL 90 min: 1273.89 ± 395.82 kcal, *p* = 0.516), breakfast calories (SJL 30 min: 355.52 ± 123.63 kcal, SJL 30–90 min: 322.87 ± 155.14 kcal, SJL > 90 min: 326.06 ± 186.27 kcal, *p* = 0.848) lunch calories (SJL < 30 min: 321.00 ± 178.13 kcal, SJL 30–90 min: 397.39 ± 243.41 kcal, SJL > 90 min: 270.44 ± 150.22, *p* = 0.140) dinner calories (SJL < 30 min: 455.33 ± 191.91 kcal, SJL 30–90 min: 493.83 ± 224.96 kcal, SJL > 90 min: 476.94 ± 234.08, *p* = 0.913)	Sample size calculation was performed, but not for SJL. Association of SJL with dietary intake not listed as study objective. Dietary intake assessed only for the previous 24 h.
Lanes et al., 2020 [25]	US	Cross-sectional	79 (23 for energy intake)	Patients with overweight and obesity, M/F	39 ± 8	Undisclosed	7 day sleep log	Time-stamped photographic food records for 3 days	The relationships among dietary quality, timing of energy intake, and sleep.	SJL was associated with a higher percentage of fat in the diet (r = 0.55, *p* = 0.007). No significant associations were found between the diet quality and timing variables.	Very small sample in the analysis on energy intake. No power calculation. Inclusion/exclusion criteria were not available
Mota et al., 2019 [27]	Brazil	Cross-sectional	792	Obesity-related chronic diseases, M/F	55.9 ± 12.4	MSF-MSW	Questionnaire	24 h food recall	The association of SJL with food consumption.	After adjustments for, patients with SJL vs. without SJL consumed more: total calories (1621.6 ± 38.1 vs. 1508.3 ± 20.2, *p* < 0.001) protein (85.1 ± 2.7 g vs. 76.5 ± 1.4 g, *p* < 0.001) total fat (59.9 ± 2.1 g vs. 52.6 ± 1.0 g, *p* = 0.002) saturated fat (19.4 ± 0.8 g vs. 17.3 ± 0.4 g, *p* = 0.01) cholesterol (315.9 ± 14.1 g vs. 246.2 ± 6.3 g, *p* < 0.001) servings per day of meat and eggs (2.5 ± 0.10 vs. 2.2 ± 0.05, *p* = 0.002) servings per day of sweets (1.7 ± 0.12 vs. 1.4 ± 0.06, *p* = 0.04) In the logistic regression SJL was associated with a higher risk of consumption above recommendations for total fat (OR = 1.3, 95% CI: 1.1–1.9; *p* = 0.03), saturated fat (OR = 1.2, 95% CI: 1.1–2.0; *p* = 0.01) and cholesterol (OR = 1.8, 95% CI: 1.3–2.6; *p* < 0.001), independent of age, sex, BMI, minutes of physical activity per week, mean sleeping duration and time since diagnosis of T2 D.	No power calculation. Dietary intake assessed only for the previous 24 h.
Zeron-Rugerio et al., 2019 [32]	Spain	Cross-sectional	534	Healthy young adults, M/F	18–25	Time in bed in weekends—time in bed during weekdays	Questionnaire	Mediterranean Diet Quality Index for children and adolescents (KIDMED)	Association of SJL with the adherence to the Mediterranean diet.	Subjects with higher SJL had a lower adherence to Mediterranean diet (β = −0.305, 95% CI: −0.503–−0.107, *p* = 0.003) independent of age, gender, physical activity, and sleep duration. SJL was associated with a lower intake of fresh or cooked vegetables (less than once a day, *p* < 0.05) and skipping breakfast (p < 0.05) independent of age, gender, and physical activity.	Young, healthy, and highly educated participants. No power calculation.
Almoosawi et al., 2018 [35]	UK	Cross-sectional	2433	General population, M/F	19–64	Difference in sleep duration between weekends and weekdays	Questionnaire for the past 7 days	Food diary	Association of sleep duration and SJL with dietary patterns.	An inversed u-shaped nonlinear association between SJL and the healthy dietary pattern was observed. Scores for healthy dietary pattern increased up to a SJL duration of 1 h 45 min. Beyond this point the scores declined, showing an inverse association of SJL duration with healthy dietary pattern. This association was independent of potential covariates, including BMI (β for quadratic term = −0.03, 95% CI: −0.04, −0.01, *p* = 0.006).	No power calculation for the objective of the analysis.
Zhang et al., 2018 [22]	China	Cross-sectional	977	Young adults, M/F	<19 to >21	MSF-MSW	MCTQ	Questionnaire on the frequency of stimulant drink consumption	The association between SJL, sleep duration, chronotype and stimulant consumption.	No association of SJL with a higher likelihood of sugar-sweetened beverage consumption was observed in the model adjusted for age, sex, race, grade, the type of university, mother’s education, father’s education, outdoor activities on workdays or free days and average sleep duration (OR = 0.93, 95% CI: 0.72–1.20).	Young, and highly educated participants. No sample size/power calculation.
Polugrudov et al., 2017 [24]	Russia	Cross-sectional, experimental	66	Healthy normal weight young participants, M/F	23.2 ± 3.4	MSF-MSW	MCTQ	VAS for appetite	The impact of SJL on appetite in response to a meal.	Similar caloric intake at test meal in SJL groups (SJL < 1 h: 923.1 ± 77.3 kcal; SJL 1–2 h: 1144.6 ± 79.3 kcal; SJL > 2 h: 1122.1 ± 82.7 kcal). Despite similar caloric intake participants with SJL had higher ratings of hunger and prospective food intake after meal than in the SJL ≤ 1 h group. Post meal mean SQ (mean value of the SQs for hunger, prospective food intake, satiety, and fullness) was significantly lower in participants in the 1 < SJL ≤ 2 h and SJL > 2 h groups (1.3 and 1.7 times, respectively) compared to those in the SJL ≤ 1 h group (*p* < 0.010). No association of SJL with the desire to eat sweets, salty, fatty, or savoury foods was observed.	No power calculation. Small sample. Young, healthy, highly educated, and normal weight participants.
Potter et al., 2017 [23]	UK	Cross-sectional	72	General population, M/F	43.5 ± 16.6	Undisclosed	MCTQ	24 h food recall	Association of chronotype and SJL with diet composition, metabolic outcomes, and physical activity.	1 h increase in SJL was associated with 20 g lower consumption of sugar (95% CI −33 to −6 g, *p* = 0.004) after adjustment for age, chronotype, ethnicity, and sex.	No power calculation. Small sample. Inclusion/exclusion criteria were not available.
Silva et al., 2016 [29]	Brazil	Cross-sectional	204	Healthy young participants, M/F	18–39	MSF-MSW	Questionnaire	70-item FFQ	The relationship between chronotype, SJL, perceived sleep debt and dietary intake.	SJL was negatively associated with servings per day of beans (β = −0.16; *p* = 0.02), independent of age, BMI, and sex.	No power calculation. Young, healthy, and highly educated participants.

SJL = social jetlag; SJLsc = social jetlag sleep corrected; MCTQ = Munich Chronotype Questionnaire; FFQ = food frequency questionnaire; BMI = body mass index; MSF = mid sleep time during free days; MSW = mid sleep time during week days; CI = confidence interval; OR = odds ratio.

**Table 2:** Studies reporting the association of social jetlag with meal timing.

Authors, Year	Country	Study Design	Population Sample	Population Type, Gender	Age, Years	SJL Calculation	SJL Assessment Method	Meal Timing Assessment Method	Main Study Objective	Main Findings	Limitations
Bodur et al., 2021 [19]	Turkey	Cross-sectional	710	General population, M/F	19.0–24.0	MSF-MSW	Questionnaire for the previous night	24 h food recall	Energy, macronutrient intake and diet qualities in persons with and without SJL during working vs. non-working days.	Delayed meal timing in free days compared to workdays irrespective of SJL presence. Significant difference in the change of breakfast time in those with SJL compared to those without SJL (02:19 vs. 01:29, *p* < 0.001). A similar eating window was observed in both groups during free and workdays. It decreased significantly during free days in participants with SJL (−01:47 vs. −01:45, *p* < 0.001) resulting in a shorter eating window in free days in those with SJL as compared to those without SJL Shorter eating window in free days in those with SJL as compared to those without SJL (8:42 vs. 9:07).	Dietary intake assessed for the 24 h prior to the study visits. Sleep mid-point assessed only for the night before interview. No power calculation.
Rusu et al., 2021 [28]	Romania	Cross-sectional	80	Healthy normal weight young participants, M/F	31.7 ± 6.7	MSF-MSW	Questionnaire for the previous month	24 h food recall VAS for food preferences	Effect of SJL on perceived appetite, hunger, and ghrelin in healthy subjects in free-living conditions.	A later lunch and dinner time in group with SJL as compared to group without SJL (14:03 h vs. 13:46 h and 20:00 h vs. 19:38 h, respectively). A higher frequency of snacking after dinner (35.0% vs. 30.0%) and a later time of this snack was observed in participants with SJL than in those without SJL.	Dietary intake assessed only for the previous 24 h. No power calculation. Small sample. Statistical significance of differences between groups not tested. Young, healthy, highly educated, and normal weight participants.
Mota et al., 2019 [27]	Brazil	Cross-sectional	792	Obesity-related chronic diseases, M/F	55.9 ± 12.4	MSF-MSW	Questionnaire	24 h food recall	The association of SJL with food consumption.	Patients with SJL had breakfast (7:44 ± 0:04 vs. 7:20 ± 0:02, *p* = 0.02), early afternoon snack (15:54 ± 0:05 vs. 15:36 ± 0:02, *p* < 0.001) and dinner time (20:12 ± 0:11 vs. 19:42 ± 0:06, *p* = 0.01) later than those without SJL. Patients with SJL reported a longer eating window (13:09 ± 0:10 vs. 12:45 ± 0:06, *p* = 0.03), higher calorie intake after 9 p.m. (188.4 ± 26.9 vs. 105.1 ± 10.2, *p* < 0.001), and a higher proportion of calories consumed after 9 p.m. (10.5 ± 0.9% vs. 6.2 ± 0.5%, *p* < 0.001) than those without SJL.	No power calculation. Dietary intake assessed only for the previous 24 h.
Zeron Rugerio et al., 2019 [33]	Spain Mexico	Cross-sectional	1106	Healthy young participants, M/F	18–25	MSF-MSW	MCTQ	Questionnaire	The association of eating jet lag with BMI.	SJL was associated with variability in meal timing (breakfast jetlag β = 0.720, 95% CI: 0.640–0.800, *p* < 0.00001; lunch jetlag β = 0.049, 95% CI: 0.002–0.096, *p* = 0.042; dinner jetlag β = 0.072, 95% CI: 0.038–0.110, *p* < 0.001), and eating jet lag (β = 0.270, 95% CI: 0.210–0.330, *p* < 0.001) independent of age, gender, nationality, diet quality, sleep duration, and physical activity.	No power calculation. Young healthy and highly educated participants.
Anothaisintawee et al., 2018 [21]	Thailand	Cross-sectional	2133	Patients with prediabetes, M/F	32–92	MSF-MSW	Questionnaire	24 h food recall	The contribution of chronotype to body mass index	A higher SJL was associated with later dinner time.	Association of SJL with meal timing was an incidental finding. No power calculation.
Silva et al., 2016 [29]	Brazil	Cross-sectional	204	Healthy young participants, M/F	18–39	MSF-MSW	Questionnaire	70-item FFQ	The relationship between chronotype, SJL, perceived sleep debt and dietary intake.	A weak negative correlation between SJL and breakfast time (r = −0.23, *p* < 0.01) was observed, and this correlation was independent of age.	No power calculation. Young, healthy, and highly educated participants.

SJL = social jetlag; MCTQ = Munich Chronotype Questionnaire; MSF = mid sleep time during free days; MSW = mid sleep time during week days; FFQ = food frequency questionnaire; VAS = visual analogue scale; BMI =body mass index; CI = confidence interval.

**Table 3:** Studies reporting the association of day-to-day variability in sleep timing with dietary intake and meal timing.

Authors, Year	Country	Study Design	Population Sample	Population Type, Gender	Age, Years	Metric Used for Sleep Timing Variability	Day-to-Day Sleep Timing Variability Assessment Method	Dietary Intake Assessment Method	Main Study Objective	Main Findings	**Limitations**
Association of Day-to-Day Variability in Sleep Timing with Dietary Intake											
Hooker et al., 2020 [18]	US	Cross-sectional	103	General population, M/F	18–50	StDev	7 days of sleep actigraphy Sleep diaries	7 days of food diaries	To evaluate the characteristics of health behavior variabilities for 3 key health behaviours: physical activity, caloric intake, and sleep (duration and timing).	No significant correlation of variability in sleep timing with average caloric intake (Pearson correlation coefficient = −0.14, *p* > 0.05) or variability in caloric intake (Pearson correlation coefficient = −0.10, *p* > 0.05) was found.	No power calculation. Young, healthy, highly educated, and highly active participants.
Duncan et al., 2016 [19]	Australia	Cross-sectional	1317	General population, M/F	57 (46, 66)	Weighted average	Sleep Timing Questionnaire	13-item Diet Quality Tool	To examine the relationship of bedtime, rise-time, and variation in these with dietary behaviours, physical activity, alcohol consumption, and partial sleep deprivation.	Bed-time variability was negatively associated with dietary quality (β = 0.96, 95% CI: 0.93–0.99, *p* = 0.002), independent of waking time variability, usual bedtime and waking time, age, gender, smoking status, number of health conditions, work schedule, BMI, and days of insufficient sleep.	No power calculation. Median age was 57 and thus participants may have a regular sleep pattern.
Chan, 2018 [34]	US	Cross-sectional	78	Healthy young adults, M/F	18–30	Not disclosed	7 days of sleep actigraphy	7 days of food diaries	To evaluate the associations of actigraphy-assessed sleep with high palatable food consumption in free-living conditions.	Bedtime variability > 90 min was associated with less frequently high palatable food consumption at breakfast than in those with bedtime variability < 90 min (OR 2.31 vs. 3.78, *p* = 0.004). There was no difference at lunch and dinner.	No power calculation. Young, healthy participants.
Yamaguchi, 2013 [36]	Japan	Cross-sectional	1368	General population, M/F	35–69	Good/poor based on answers	Questions on sleep onset/offset regularity for the previous year	47-item FFQ	To evaluate the associations between dietary factors and sleep–wake regularity.	Lowest quartile of protein intake (<10.8% energy) was associated with poor sleep-wake regularity (OR = 2.1; 95% CI: 1.3–3.3). The highest quartile of carbohydrate intake (≥70.7% energy) was associated with poor sleep-wake regularity (OR = 2.1; 95% CI: 1.3–3.5). The lowest intake of the staple foods at breakfast and the highest intake at lunch and dinner were associated with poor sleep-wake regularity (OR = 2.5, 95% CI: 1.7–3.5 for breakfast, OR = 3.2, 95% CI: 1.4–7.2 and 2.5, 95% CI: 1.3–4.9 for lunch and dinner, respectively).	No power calculation. Sleep–wake regularity self-reported for the past year.
Association of day-to-day variability in sleep timing with meal timing											
Linnaranta, 2020 [37]	Canada	Cross-sectional	29	Patients with eating disorders, F	26 (9.75)	StDev of centre of daily inactivity	14 days of actigraphy	14 days of food diaries	To correlate eating patterns with sleep.	Variability in sleep timing was associated with meal timing variability (rho = 0.47, *p* = 0.010).	No power calculation. Patients with eating disorders.

BMI = body mass index; FFQ = food frequency questionnaire; CI = confidence interval; OR = odds ratio; StDev = standard deviation.

## Data Availability

Not applicable.

## Appendix A

Search queries used.

### Appendix A.1. PubMed

- (“eating”[MeSH Terms] OR “eating”[All Fields] OR (“food”[All Fields] AND “intake”[All Fields]) OR “food intake”[All Fields]) AND ((“social behavior”[MeSH Terms] OR (“social”[All Fields] AND “behavior”[All Fields]) OR “social behavior”[All Fields] OR “sociality”[All Fields] OR “social”[All Fields] OR “socialisation”[All Fields] OR “socialization”[MeSH Terms] OR “socialization”[All Fields] OR “socialise”[All Fields] OR “socialised”[All Fields] OR “socialising”[All Fields] OR “socialities”[All Fields] OR “socializations”[All Fields] OR “socialize”[All Fields] OR “socialized”[All Fields] OR “socializers”[All Fields] OR “socializes”[All Fields] OR “socializing”[All Fields] OR “socially”[All Fields] OR “socials”[All Fields]) AND (“jet lag syndrome”[MeSH Terms] OR (“jet”[All Fields] AND “lag”[All Fields] AND “syndrome”[All Fields]) OR “jet lag syndrome”[All Fields] OR “jetlag”[All Fields]))
- (“eating”[MeSH Terms] OR “eating”[All Fields] OR (“dietary”[All Fields] AND “intake”[All Fields]) OR “dietary intake”[All Fields]) AND ((“social behavior”[MeSH Terms] OR (“social”[All Fields] AND “behavior”[All Fields]) OR “social behavior”[All Fields] OR “sociality”[All Fields] OR “social”[All Fields] OR “socialisation”[All Fields] OR “socialization”[MeSH Terms] OR “socialization”[All Fields] OR “socialise”[All Fields] OR “socialised”[All Fields] OR “socialising”[All Fields] OR “socialities”[All Fields] OR “socializations”[All Fields] OR “socialize”[All Fields] OR “socialized”[All Fields] OR “socializers”[All Fields] OR “socializes”[All Fields] OR “socializing”[All Fields] OR “socially”[All Fields] OR “socials”[All Fields]) AND (“jet lag syndrome”[MeSH Terms] OR (“jet”[All Fields] AND “lag”[All Fields] AND “syndrome”[All Fields]) OR “jet lag syndrome”[All Fields] OR “jetlag”[All Fields]))
- (“sleep”[MeSH Terms] OR “sleep”[All Fields] OR “sleeping”[All Fields] OR “sleeps”[All Fields] OR “sleep s”[All Fields]) AND (“variabilities”[All Fields] OR “variability”[All Fields] OR “variable”[All Fields] OR “variable s”[All Fields] OR “variables”[All Fields] OR “variably”[All Fields]) AND (“eating”[MeSH Terms] OR “eating”[All Fields] OR (“dietary”[All Fields] AND “intake”[All Fields]) OR “dietary intake”[All Fields])
- (“sleep”[MeSH Terms] OR “sleep”[All Fields] OR “sleeping”[All Fields] OR “sleeps”[All Fields] OR “sleep s”[All Fields]) AND (“variabilities”[All Fields] OR “variability”[All Fields] OR “variable”[All Fields] OR “variable s”[All Fields] OR “variables”[All Fields] OR “variably”[All Fields]) AND (“eating”[MeSH Terms] OR “eating”[All Fields] OR (“food”[All Fields] AND “intake”[All Fields]) OR “food intake”[All Fields])
- (“eating”[MeSH Terms] OR “eating”[All Fields] OR (“dietary”[All Fields] AND “intake”[All Fields]) OR “dietary intake”[All Fields]) AND ((“regular”[All Fields] OR “regularities”[All Fields] OR “regularity”[All Fields] OR “regularization”[All Fields] OR “regularizations”[All Fields] OR “regularize”[All Fields] OR “regularized”[All Fields] OR “regularizer”[All Fields] OR “regularizers”[All Fields] OR “regularizes”[All Fields] OR “regularizing”[All Fields] OR “regulars”[All Fields]) AND (“sleep”[MeSH Terms] OR “sleep”[All Fields] OR “sleeping”[All Fields] OR “sleeps”[All Fields] OR “sleep s”[All Fields]))
- ((“eating”[MeSH Terms] OR “eating”[All Fields] OR (“food”[All Fields] AND “intake”[All Fields]) OR “food intake”[All Fields]) AND ((“regular”[All Fields] OR “regularities”[All Fields] OR “regularity”[All Fields] OR “regularization”[All Fields] OR “regularizations”[All Fields] OR “regularize”[All Fields] OR “regularized”[All Fields] OR “regularizer”[All Fields] OR “regularizers”[All Fields] OR “regularizes”[All Fields] OR “regularizing”[All Fields] OR “regulars”[All Fields]) AND (“sleep”[MeSH Terms] OR “sleep”[All Fields] OR “sleeping”[All Fields] OR “sleeps”[All Fields] OR “sleep s”[All Fields])))

### Appendix A.2. Embase

- (‘social jetlag’/exp OR ‘social jetlag’) AND (‘food intake’/exp OR ‘food intake’) AND [<1966–2021]/py
- (‘social jetlag’/exp OR ‘social jetlag’) AND (‘dietary intake’/exp OR ‘dietary intake’) AND [<1966–2021]/py
- (‘sleep’/exp OR sleep) AND (‘variability’/exp OR variability) AND (‘food intake’/exp OR ‘food intake’) AND [<1966–2021]/py
- (‘sleep’/exp OR sleep) AND (‘variability’/exp OR variability) AND (‘dietary intake’/exp OR ‘dietary intake’) AND [<1966–2021]/py
- (‘sleep’/exp OR ‘sleep’) AND ‘regularity’ AND (‘food intake’/exp OR ‘food intake’) AND [<1966–2021]/py
- (‘sleep’/exp OR ‘sleep’) AND ‘regularity’ AND (‘dietary intake’/exp OR ‘dietary intake’) AND [<1966–2021]/py

### Appendix A.3. Clarivate Analytics Web of Science

- social jetlag (All Fields) AND food intake (All Fields)
- social jetlag (All Fields) AND dietary intake (All Fields)
- sleep (All Fields) AND variability (All Fields) AND food intake (All Fields)
- sleep (All Fields) AND variability (All Fields) AND dietary intake (All Fields)
- sleep (All Fields) and regularity (All Fields) and food intake (All Fields)
- sleep (All Fields) and regularity (All Fields) and dietary intake (All Fields)
